# The predictive value of free thyroxine combined with tubular atrophy/interstitial fibrosis for poor prognosis in patients with IgA nephropathy

**DOI:** 10.3389/fendo.2024.1372824

**Published:** 2024-05-14

**Authors:** Bixia Yang, Wen Zhou, Liqin Cui, Li Tian, Yanhong Ni, Min Yang, Yan Yang

**Affiliations:** ^1^ Department of Nephrology, The Third Affiliated Hospital of Soochow University, Changzhou, China; ^2^ Changzhou Medical Center, Nanjing Medical University, Changzhou, China

**Keywords:** IgA nephropathy, free thyroxine (FT4), tubular atrophy/interstitial fibrosis, renal prognosis, renal progression

## Abstract

**Background:**

IgA nephropathy (IgAN), the most common type of glomerulonephritis, has great individual differences in prognosis. Many studies showed the relationship between thyroid hormones and chronic kidney disease. However, the relationship between free thyroxine (FT4), as a thyroid hormone, and IgAN is still unclear. This study aimed to evaluate the impact of FT4 on IgAN prognosis.

**Methods:**

This retrospective study involved 223 patients with biopsy-proven IgAN. The renal composite outcomes were defined as: (1) ESRD, defined as eGFR < 15 ml/(min·1.73 m^2^) or initiation of renal replacement therapy (hemodialysis, peritoneal dialysis, renal transplantation); (2) serum creatinine doubled from baseline; (3) eGFR decreased by more than 50% from baseline. The predictive value was determined by the area under the curve (AUC). Kaplan-Meier and Cox proportional hazards analyses assessed renal progression and prognosis.

**Results:**

After 38 (26–54) months of follow-up, 23 patients (10.3%) experienced renal composite outcomes. Kaplan-Meier survival curve analysis showed that the renal survival rate of the IgAN patients with FT4<15.18pmol/L was lower than that with FT4≥15.18pmol/L (P < 0. 001). Multivariate Cox regression model analysis showed that FT4 was a protective factor for poor prognosis of IgAN patients, whether as a continuous variable or a categorical variable (HR 0.68, 95%CI 0.51–0.90, P =0.007; HR 0.04, 95%CI 0.01–0.20, P <0.001). ROC curve analysis showed that FT4 combined with t score had a high predictive value for poor prognosis of IgAN patients (AUC=0.881, P<0.001).

**Conclusion:**

FT4 was a protective factor for IgAN. In addition, FT4 combined with tubular atrophy/interstitial fibrosis had a high predictive value for poor prognosis of IgAN.

## Introduction

IgA nephropathy (IgAN) is the most prevalent primary glomerulonephritis worldwide and carries a considerable lifetime risk of renal failure (1). 20%-50% of patients develop end-stage renal disease (ESRD) approximately 20 years after diagnosis (2–4). The prognosis of IgAN varies greatly, and the prognosis of individual patients remains difficult to predict. More indicators are urgently needed to predict the prognosis of IgAN patients to improve treatment strategies.

The thyroid gland interacts with the kidney. Thyroid hormone can directly affect renal growth and development, hemodynamics, and electrolyte balance (5). The kidney is also involved in thyroid hormone metabolism and elimination. In addition, IgAN and some thyroid diseases are affected by autoimmunity (6, 7). Free thyroxine is an important member of the thyroid hormone, but its effect on IgAN has not been studied.

In this study, we explored the effect of free thyroxine (FT4) on the prognosis of IgAN for the first time. Additionally, we demonstrated the predictive value of FT4 combined with renal tubular atrophy/interstitial fibrosis for the poor prognosis of IgAN.

## Materials and methods

### Patients

We retrospectively analyzed the clinical data of patients with primary IgAN who were admitted to the First People’s Hospital of Changzhou from January 1, 2018 to December 31, 2021. Renal biopsies were performed on all these patients. Inclusion criteria were as follows: (1) Estimated glomerular filtration rate (eGFR) > 15 ml/(min·1.73 m ^2^) during renal biopsy; (2) Complete baseline data. Exclusion criteria were as follows: (1) lupus nephritis, Henoch-Schonlein purpura nephritis, hepatitis B virus-related nephritis and other secondary IgAN patients; (2) complicated with other primary glomerular diseases; (3) complicated with other infectious diseases, malignant tumors or serious organic diseases. (4) loss of follow-up or death due to non-renal causes. Loss of follow-up was defined as no follow-up record at our hospital after renal puncture. This study was approved by the Ethics Committee of the First People’s Hospital of Changzhou (2024 #11).

### Clinical data

The general data and laboratory test results of the patients at the time of IgAN diagnosis were collected from the clinical medical record system as baseline data. Gender, age, proportion of hypertension, body mass index(BMI), hemoglobin, albumin, globulin,TSH, free triiodothyronine (FT3), FT4,Thyroid disease and treatment, serum creatinine (Scr), uric acid, 24h urine protein, urine red blood cell count (URBC) and treatment methods were collected. The eGFR was calculated using the Chronic Kidney Disease Epidemiology Collaboration (CKD-EPI) equation (**Supplementary Table 1**). Hypertension was defined as SBP≥140 mmHg and/or DBP≥90 mmHg. Anemia was defined as hemoglobin concentration <120g/L for men and <110g/L for women. Hyperuricemia was defined as uric acid concentration >420µmol/L in males and > 360µmol/L in females. Hypoalbuminemia was defined as serum albumin <30g/L.

### Pathological data

All renal biopsy specimens were evaluated by experienced pathologists and nephrologists who were blinded to the patients’ information. The updated Oxford classification of IgAN (8) we used for the evaluation of pathological lesions in biopsy specimens was as follows: (1) mesangial hypercellularity(M): more than 3 mesangial cells in more than 50% of the mesangial area was defined as M1, otherwise M0; (2) endocapillary hypercellularity (E): hypercellularity in the lumen of glomerular capillaries was defined as E1, otherwise E0; (3) segmental glomerulosclerosis (S): any variable degree of loop involvement, including segmental sclerosis of the glomerulus, was defined as S1,otherwise S0; (4) tubular atrophy and interstitial fibrosis (T): lesion size ≤ 25% was defined as T0, 26%-50% as T1, and > 50% as T2; (5) cellular or fibrocellular crescents (C): no crescent was defined as C0, < 25% as C1, and≥25% as C2.

### Follow-up

Follow-up was performed through the hospital medical record system or by telephone. The follow-up period ended in August 2023. The renal composite outcomes were defined as: (1) ESRD, defined as eGFR < 15 ml/(min·1.73 m^2^) or initiation of renal replacement therapy (hemodialysis, peritoneal dialysis, renal transplantation); (2) serum creatinine doubled from baseline; (3) eGFR decreased by more than 50% from baseline.

### Statistical analysis

We used SPSS 26.0 to analyze all data. Quantitative variables with normal distribution were expressed mean ± SD, and comparison between groups use independent sample t-test; Non-normal distribution quantitative variables were represented as median and interquartile range (IQR), and Mann-Whitney U test was used for comparison between groups. Categorical data were expressed as frequencies (percentages), and comparison between groups was analyzed using the chi-square test or Mann-Whitney U test. Kaplan-Meier survival curves were drawn to compare the renal survival rates of the two groups. Univariate and multivariate Cox regression models were used to analyze the risk factors affecting renal composite outcomes in IgAN patients. The multivariate Cox regression analysis included the variables with P-value<0.05 in univariate Cox regression analysis. ROC curve was used to analyze the predictive efficacy of different indicators for renal composite outcomes in IgAN patients. The predictive value was determined by the AUC. P < 0. 05 was considered statistically significant. In addition, sensitivity analyses were performed by excluding patients with thyroid diseases and thyroid hormone replacement therapy.

## Results

### Predictive efficacy of FT4 for renal composite outcomes in IgAN patients

**Figure 1:**
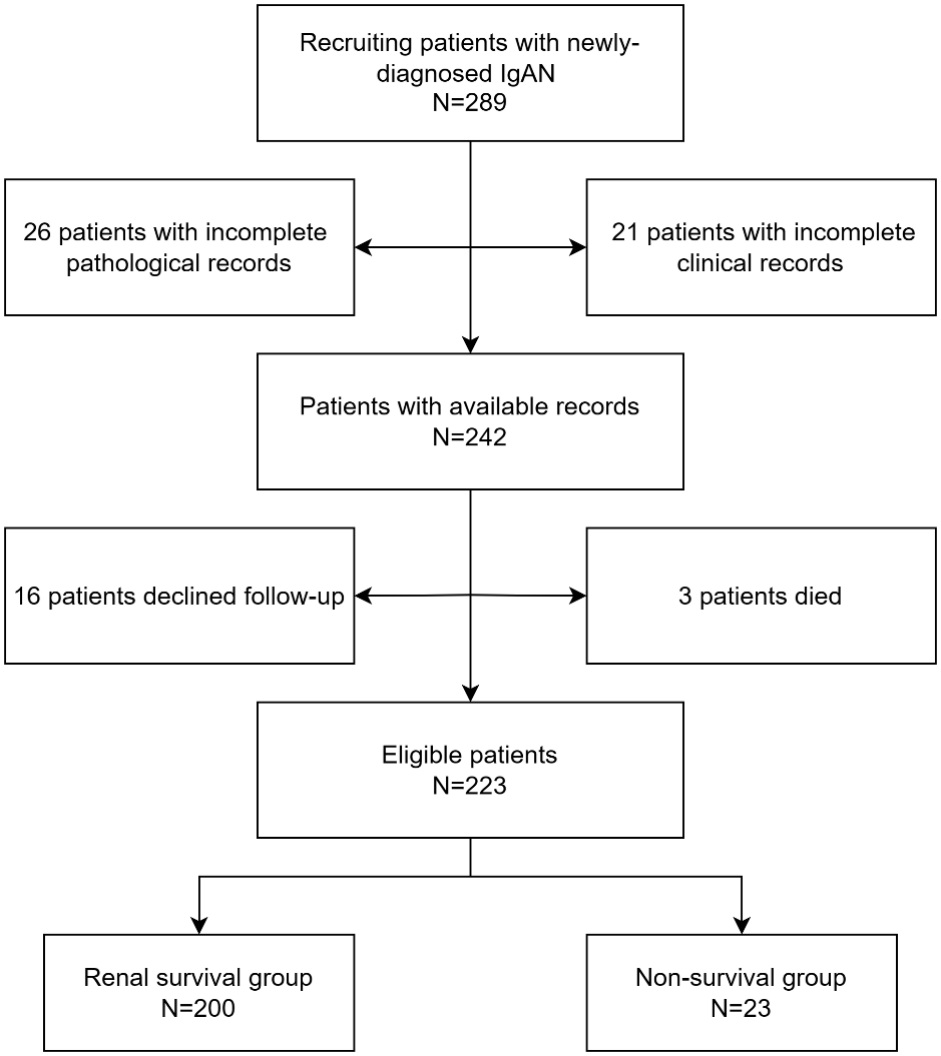
Patients screening flowchart.

**Figure 2:**
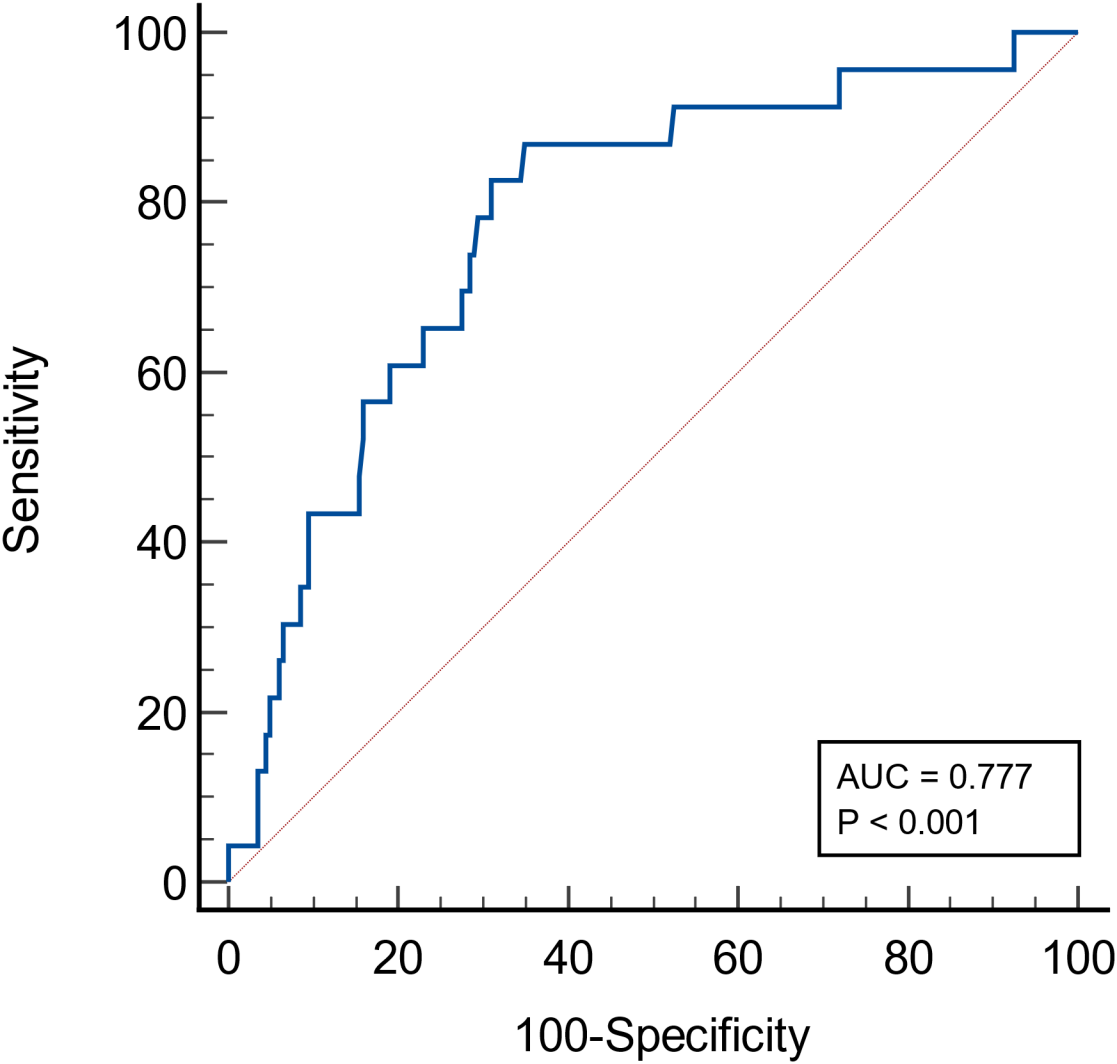
ROC curve of the probability of FT4 in predicting renal composite outcomes in patients with IgA nephropathy.

A total of 223 IgAN patients were enrolled (**Figure 1**). The results of ROC curve analysis showed that the best cut-off value of FT4 for predicting renal composite outcomes in IgAN patients was 15.18 pmol/L. The AUC of FT4 for predicting renal composite outcomes in IgAN patients was 0. 777, and the sensitivity and specificity were 87.0% and 65.0%, respectively (**Figure 2**).

### Baseline clinical characteristics of patients with IgAN

**Table 1** described the baseline characteristics of 223 IgAN patients at the time of renal biopsy. Their median age was 39 (30–50) years, and 123 (55.2%) patients were male. 88 (39.5%) patients had hypertension. The median serum creatinine at renal biopsy was 94 (70–121) µmol/l, and the median 24h urine protein was 2 (1.13–3.43) g/day. 8 (3.6%) patients had hypothyroidism, 17 (7.6%) patients had subclinical hypothyroidism, 5 (2.2%) patients had low T3 syndrome and one (0.5%) patient had subclinical hyperthyroidism. 4 (1.8%) patients were treated with supplemental thyroid hormone, of whom three had hypothyroidism, and one had subclinical hypothyroidism. Compared with the renal survival group, the non-renal survival group had lower levels of FT3 and FT4, and higher levels of serum creatinine and 24-hour urinary protein. In addition, the non-renal survival group had significantly higher proportion of anemia, hypoalbuminemia, and hyperuricemia (56.5% vs. 12.5%, P<0.001; 39.1% vs 16.0%, P=0.015; 69.6% vs. 44.0%, P=0.020), but lower proportion of ACEI/ARB using to reduce urinary protein (26.1 vs. 55.5%, P=0.007).

**Table 1:** Baseline clinical characteristics in IgAN patients.

Clinical characteristics	Total (223)	Renal survival group (200)	Non-renal survival group (23)	P-value
Male (n, %)	123(55.2)	107(53.5)	16(69.6)	0.142
Age(years) *	39 (30, 50)	38(30, 48.5)	40(31.5, 51.25)	0.377
Time(months) *	38 (26, 54)	41(25, 54.5)	30(23.75, 43)	0.016
BMI (kg/m2)	23.93 ± 3.64	23.88 ± 3.63	24.32 ± 3.81	0.577
Hypertension (n, %)	88(39.5)	75(37.5)	13(56.5)	0.077
Anemia (n, %)	38 (17)	25(12.5)	13(56.5)	<0.001
Hypoalbuminemia (n, %)	41(18.4)	32(16.0)	9(39.1)	0.015
GLB(g/L) *	26.1(23.9, 28.8)	26.3(24.05, 28.85)	24.9(21.38, 29.08)	0.129
Hyperuricemia (n, %)	104(46.6)	88(44.0)	16(69.6)	0.020
Scr(µmol/L) *	94 (70, 121)	90(68, 111.4)	187.50(124.75, 236.75)	<0.001
eGFR (ml/min/1.73m^2^)	82.18(57.09, 102.42)	85.75(65.50, 104.07)	39.06(28.64, 48.63)	<0.001
24h urinary protein (g/L) *	2 (1.13, 3.43)	1.85(1.10, 2.83)	3.65(1.94, 6.58)	0.001
URBC *	63.9(18.6, 146.1)	63.8(17.9, 143.7)	114.25(26.55, 249.63)	0.308
TSH (uIU/ml) *	2.55(1.75,3.88)	2.61(1.68, 3.86)	2.32(1.09, 4.19)	0.856
FT3 (pmol/L)	4.41 ± 0.76	4.48 ± 0.74	3.84 ± 0.61	<0.001
FT4 (pmol/L)	15.66 ± 2.62	15.93 ± 2.47	13.34 ± 2.79	<0.001
FT4(<15.18 pmol/L)	90(40.4)	70(35.0)	20(87.0)	<0.001
treatment				
ACEI/ARB (n, %)	117(52.5)	111(55.5)	6(26.1)	0.007
Prednisone or other immunosuppressive agents (n, %)	136(61.0)	119(59.5)	17(73.9)	0.180
Thyroid diseases				0.751
hypothyroidism	8 (3.6)	5 (2.5)	3 (13.0)	
subclinical hypothyroidism	17 (7.6)	16 (8.0)	1 (4.3)	
low T3 syndrome	5 (2.2)	5 (2.5)	0	
subclinical hyperthyroidism	1 (0.4)	1 (0.5)	0	
Thyroid hormone replacement therapy (%)	4 (1.8)	3 (1.5)	1 (4.3)	0.355

The eGFR is calculated according to the CKD-EPI formula. BMI, body mass index; GLB, globumin; Scr, serum creatinine; eGFR, estimated glomerular filtration rate; TSH, thyroid stimulating hormone; FT3, free triiodothyronine; FT4, free thyroxine; URBC, urine red blood cell; ACEI/ARB, angiotension converting enzyme inhibitors/angiotonin receptor blocker. * median (the interquartile range).

### Pathological characteristics of patients with IgAN

There were significant differences in the proportion of tubular atrophy/interstitial fibrosis and cellular/fibrous crescents between the two groups. The proportion of T2 and C2 in the non-renal survival group was significantly higher than that in the renal survival group (52.2% vs 6.0%, P<0.001; 17.4% vs. 2.0%, P=0.011). Mesangial cell proliferation, segmental glomerulosclerosis, and endocapillary cell proliferation were not significantly different between the two groups (**Table 2**).

**Table 2:** Baseline renal pathological injury score in IgAN patients.

Renal pathology	Total (N=223)	Renal survival group (200)	Non-renal survival group (23)	P-value
Oxford MEST-C, n (%)				
M				1.000
M0	8 (3.6)	8 (4.0)	0 (0)	
M1	215 (96.4)	192 (96.0)	23 (100)	
E				0.378
E0	184 (82.5)	163 (81.5)	21 (91.3)	
E1	39 (17.5)	37 (18.5)	2 (8.7)	
S				0.419
S0	75 (33.6)	69 (34.5)	6 (26.1)	
S1	148 (66.4)	131 (65.5)	17 (73.9)	
T				<0.001*
T0	157 (70.4)	151 (75.5)	6 (26.1)	
T1	42 (18.8)	37 (18.5)	5 (21.7)	
T2	24 (10.8)	12 (6.0)	12 (52.2)	
C				0.011*
C0	111 (49.8)	104 (52.0)	7 (30.4)	
C1	104 (46.6)	92 (46.0)	12 (52.2)	
C2	8 (3.6)	4 (2.0)	4 (17.4)	

MEST-C, M, mesangial hypercellularity; E, endocapillary hypercellularity; S, segmental glomerulosclerosis; T, interstitial fibrosis/tubular atrophy; C, crescents formation. *Mann-Whitney U test.

### Comparison of renal survival in IgAN patients with high and low FT4 levels

**Figure 3:**
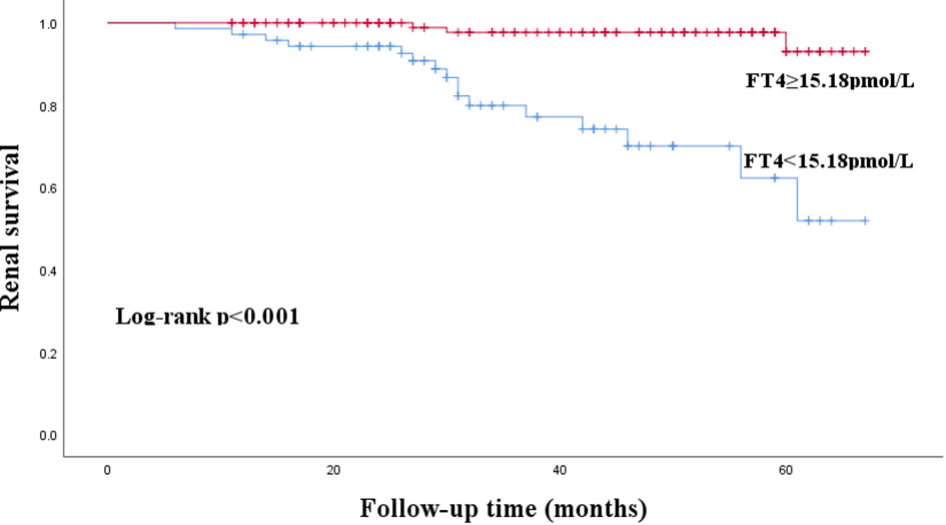
Kaplan-Meier curves for renal composite outcomes in patients with IgA nephropathy according to FT4.

The median follow-up time was 38 (26–54) months. During the follow-up period, 23 patients (10.3%) experienced renal composite outcomes. Kaplan-Meier survival curve analysis showed that the renal survival rate of the patients with FT4<15.18pmol/L was lower than that with FT4≥15.18pmol/L(P < 0. 001) (**Figure 3**).

### Analysis of risk factors for renal composite outcomes in IgAN patients

Univariate Cox regression model analysis showed that anemia (HR 8.2, 95%CI 3.57–18.84, P<0.001), hypoalbuminemia (HR 3.76, 95%CI 1.62–8.72, P=0.002), serum creatinine (HR 1.03, 95%CI 1.02–1.03, P<0.001), 24h urine protein (HR 1.14, 95%CI 1.04–1.23, P=0.003), T1 (HR 3.5, 95%CI 1.07–11.5, P=0.039), T2 (HR 28.54, 95%CI 10.08–80.75, P<0.001), C2 (HR 8.47, 95%CI 2.43–29.61, P=0.001), TSH (HR 1.08, 95%CI 1.02–1.15, P=0.015), and hypothyroidism (HR 5.46, 95%CI 1.59–18.79, P=0.007) were risk factors for renal composite outcomes in IgAN patients (P < 0. 05) (**Table 3**). Besides, FT3 (HR 0.36, 95%CI 0.23–0.58, P<0.001), FT4 (HR 0.70, 95%CI 0.60–0.81, P<0.001), FT4≥15.18pmol/L (HR 0.08, 95%CI 0.02–0.27, P<0.001), and using ACEI/ARB to treat (HR 0.33, 95%CI 0.13–0.84, P=0.020) were protective factors for IgAN patients. However, thyroid hormone replacement therapy for patients with abnormal thyroid function did not affect the prognosis of IgAN (HR 3.98, 95%CI 0.53–29.78, P=0.178).

**Table 3:** Univariate Cox analysis of renal composite outcomes in IgAN patients.

Variable	Univariate Analysis	
	HR (95% CI)	P-Value
Gender, female	0.62 (0.25–1.51)	0.292
Age, y	1.02 (0.99–1.05)	0.254
Hypertension	2.21 (0.97–5.05)	0.060
Anemia	8.2 (3.57–18.84)	<0.001
Hypoalbuminemia	3.76 (1.62–8.72)	0.002
Hyperuricemia	2.87 (1.18–6.99)	0.020
Scr, μmol/L	1.03 (1.02–1.03)	<0.001
24h urinary protein, g/L	1.14 (1.04–1.23)	0.003
T1^a^	3.5 (1.07–11.5)	0.039
T2 ^a^	28.54 (10.08–80.75)	<0.001
C1 ^b^	2.23 (0.87–5.66)	0.093
C2 ^b^	8.47 (2.43–29.61)	0.001
TSH, uIU/ml	1.08 (1.02–1.15)	0.015
FT3, pmol/L	0.36 (0.23–0.58)	<0.001
FT4, pmol/L	0.70 (0.60–0.81)	<0.001
FT4 (≥15.18 pmol/L)	0.08 (0.02–0.27)	<0.001
Thyroid diseases		
hypothyroidism	5.46 (1.59–18.79)	0.007
subclinical hypothyroidism	0.56 (0.74–4.17)	0.569
low T3 syndrome	0	0.98
subclinical hyperthyroidism	0	0.992
Thyroid hormone replacement therapy	3.98 (0.53–29.78)	0.178
ACEI/ARB	0.33 (0.13–0.84)	0.020
Prednisone or other immunosuppressive agents	1.71 (0.67–4.33)	0.262

Scr, serum creatinine; T, interstitial fibrosis/tubular atrophy; C, crescents formation; TSH, thyroid stimulating hormone; FT3, free triiodothyronine; FT4, free thyroxine; ACEI/ARB, angiotension converting enzyme inhibitors/angiotonin receptor blocker; ^a^ T0 was used as the reference; ^b^ C0 was used as the reference.

Multivariate Cox regression model analysis showed that even after adjusting these confounding factors, FT4 (HR 0.68, 95%CI 0.51–0.90, P =0.007) was still a protective factor for IgAN patients when considered as a continuous variable (**Table 4**). And the risk in IgAN patients with FT4≥15.18pmol/L is 0.04 times than that in patients with FT4<15.18pmol/L (HR 0.04, 95%CI 0.01–0.20, P <0.001) (**Table 5**). In addition, a higher serum creatinine and TSH were independently associated with renal composite outcomes in IgAN patients (HR 1.03, 95%CI 1.01–1.04, P<0.001 or HR 1.03, 95%CI 1.02–1.04, P<0.001; HR 1.08, 95%CI 1.01–1.14, P=0.021 or HR 1.12, 95%CI 1.05–1.19, P=0.001).

**Table 4:** Multivariate Cox analysis of renal composite outcomes in IgAN patients (FT4 was a continuous variable).

Variable	Multivariate Analysis	
	HR (95%CI)	P-Value
Anemia	0.56 (0.10, 3.20)	0.518
Hypoalbuminemia	2.22 (0.49, 9.99)	0.299
Hyperuricemia	2.36 (0.59, 9.50)	0.225
Scr, μmol/L	1.03 (1.01, 1.04)	<0.001
24h urinary protein, g/L	0.88 (0.72, 1.07)	0.200
ACEI/ARB	0.69 (0.22, 2.22)	0.534
T1^a^	2.72 (0.59, 12.47)	0.199
T2 ^a^	6.43 (1.19, 34.70)	0.031
C1^b^	0.74 (0.19, 2.83)	0.661
C2 ^b^	6.16 (0.73, 51.8)	0.094
TSH, uIU/ml	1.08 (1.01, 1.14)	0.021
FT3, pmol/L	1.05 (0.35, 3.17)	0.935
FT4, pmol/L	0.68 (0.51, 0.90)	0.007

Scr, serum creatinine; T, interstitial fibrosis/tubular atrophy; C, crescents formation; TSH, thyroid stimulating hormone; FT3, free triiodothyronine; FT4, free thyroxine; ACEI/ARB, angiotension converting enzyme inhibitors/angiotonin receptor blocker; ^a^ T0 was used as the reference; ^b^ C0 was used as the reference.

**Table 5:** Multivariate Cox analysis of renal composite outcomes in IgAN patients (FT4 was a categorical variable).

Variable	Multivariate Analysis	
	HR (95%CI)	P-Value
Anemia	0.56 (0.12, 2.61)	0.464
Hypoalbuminemia	2.45 (0.60, 9.94)	0.211
Hyperuricemia	3.44 (0.84, 14.09)	0.085
Scr, μmol/L	1.03 (1.02, 1.04)	<0.001
24h urinary protein, g/L	0.89 (0.72, 1.09)	0.258
ACEI/ARB	0.71 (0.22, 2.22)	0.550
T1^a^	3.31 (0.73, 14.96)	0.120
T2 ^a^	3.93 (0.92, 16.83)	0.065
C1 ^b^	0.93 (0.25, 3.29)	0.890
C2 ^b^	7.32 (0.82, 65.51)	0.075
TSH, uIU/ml	1.12 (1.05, 1.19)	0.001
FT3, pmol/L	0.84 (0.30, 2.36)	0.743
FT4 (≥15.18 pmol/L)	0.04 (0.01, 0.20)	<0.001

Scr, serum creatinine; T, interstitial fibrosis/tubular atrophy; C, crescents formation; TSH, thyroid stimulating hormone; FT3, free triiodothyronine; FT4, free thyroxine; ACEI/ARB, angiotension converting enzyme inhibitors/angiotonin receptor blocker; ^a^ T0 was used as the reference; ^b^ C0 was used as the reference.

### Predictive efficacy of each index for renal composite outcomes in IgAN patients

**Figure 4:**
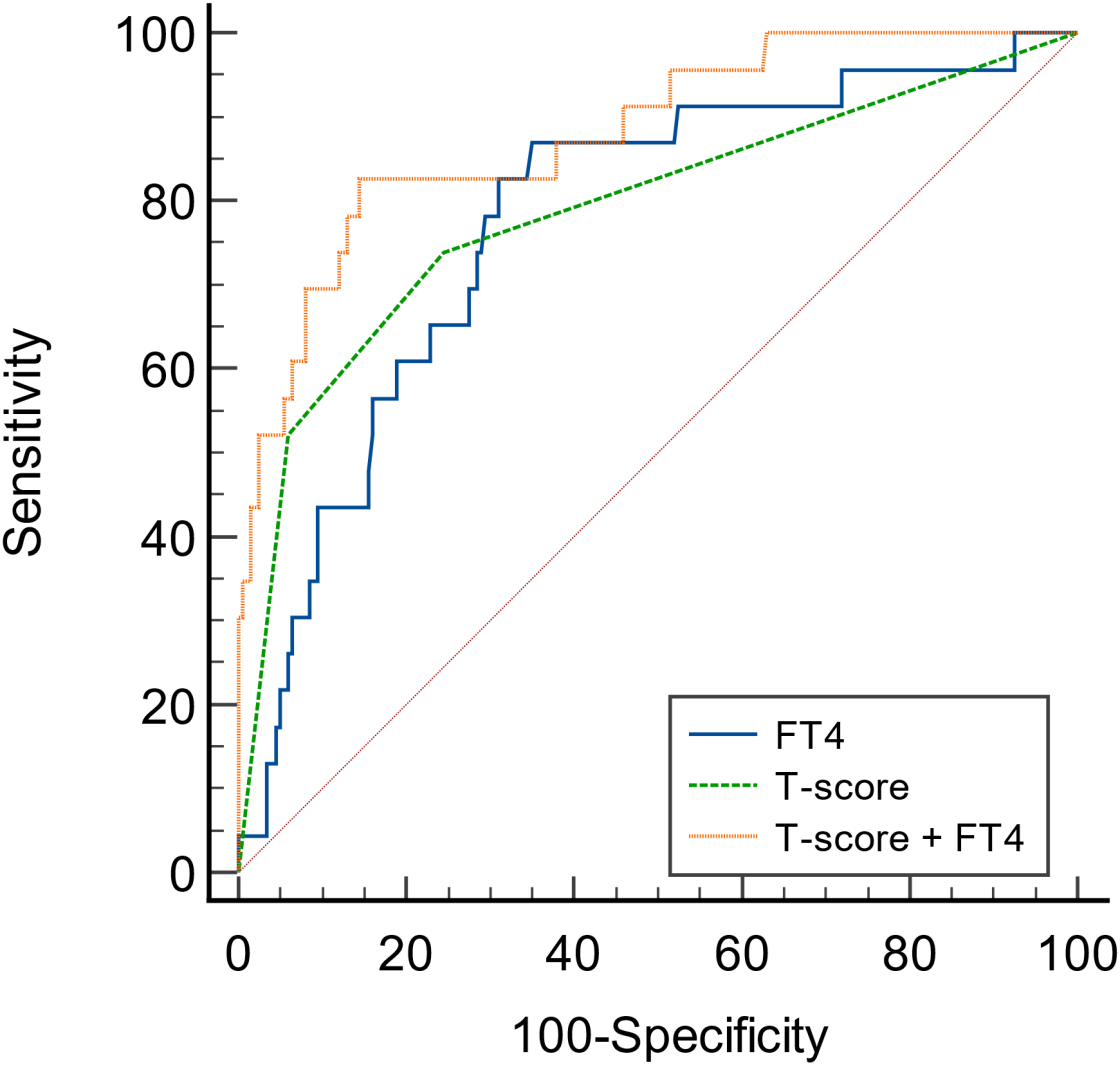
ROC curves of the probability of FT4 and T-score in predicting renal composite outcomes in patients with IgA nephropathy.

To predict the renal composite outcomes, the AUC of probability of FT4 was 0.777, with a sensitivity of 87.0% and a specificity of 65.0% (**Table 6**). The AUC of probability of T-score was 0.789, with a sensitivity of 73.9% and a specificity of 75.5%. The AUC of probability of TSH was 0.533, with a sensitivity of 43.5% and a specificity of 69.5%. The AUC of the joint probability of FT4 and T-score in predicting the renal composite outcomes was 0.881, with a sensitivity of 82.6% and a specificity of 85.5%. Moreover, it was larger than that predicted by these two indexes alone (P<0.05) (**Tables 6**, **7**, **Figure 4**).

**Table 6:** ROC curve analysis of the probability in predicting renal composite outcomes in patients with IgA nephropathy.

Variable	AUC	P-value	Sensitivity (%)	Specificity (%)
FT4	0.777	<0.001	87.0	65.0
T-score	0.789	<0.001	73.9	75.5
TSH	0.533	0.632	43.5	69.5
T-score+FT4	0.881	<0.001	82.6	85.5

FT4, free thyroxine;TSH, thyroid stimulating hormone; T-score, interstitial fibrosis/tubular atrphy score.

**Table 7:** Pairwise comparison of ROC curves.

Variable	z statistic	P-value
FT4 vs T-score	0.156	0.876
T-score+FT4 vs T-score	2.118	0.034
T-score+FT4 vs FT4	2.419	0.016

FT4, free thyroxine; T-score, interstitial fibrosis/tubular atrphy score.

### Sensitivity analyses

We conducted sensitivity analyses to determine the potential effect of confounding due to thyroid diseases and thyroid hormone replacement therapy. We excluded patients with thyroid diseases and thyroid hormone replacement therapy, and found that FT4, whether as a continuous variable or a categorical variable, was still a protective factor for the prognosis of IgAN (**Supplementary Tables 2**-**4**). Additionally, FT4 combined with T-score still had a high predictive value for poor prognosis of IgAN patients (**Supplementary Table 5**, **Supplementary Figure 1**). Therefore, thyroid diseases and thyroid hormone replacement therapy did not significantly affect the predictive performance of FT4. However, TSH was no longer an independent risk factor for the poor prognosis of IgAN patients.

## Discussion

This study demonstrated that FT4 was a protective factor and TSH was an independent risk factor for renal progression of IgAN patients, even after adjusting for covariates such as anemia, hypoalbuminemia, Hyperuricemia, 24h urinary protein, FT3, renal tubular atrophy/interstitial fibrosis, cellular or fibrocellular crescents, and treatment regimen. But, after excluding patients with thyroid disease and thyroid replacement therapy, TSH was no longer the risk factor for IgAN patients. Additionally, univariate Cox regression analysis showed that lower FT3 and hypothyroidism were associated with poor prognosis in IgAN patients. However, these associations disappeared after adjusting for confounding factors. Previous studies mainly focused on the relationship between thyroid hormones and chronic kidney disease (CKD). This study is the first to explore the relationship between thyroid hormones and the prognosis of IgAN.

Thyroid hormones can affect the function of the kidney and play a crucial role in renal physiological homeostasis. Hypothyroidism is common in CKD. Previous studies have mainly shown the relationship between hypothyroidism and renal function and its role in renal poor prognosis in CKD (9, 10). Similar to these previous studies, our study found that hypothyroidism was associated with poor prognosis in IgAN. However, most of the IgAN patients included in this study had thyroid hormone levels within the normal range. The best cut-off value of FT4 obtained by ROC curve was 15.18 pmol/L, which was higher than the FT4 value defined by hypothyroidism. Similar to the findings in CKD population, the IgAN patients with a lower FT4 level had worse prognosis. However, our results also contradict the findings of some studies. In a prospective study, higher levels of FT4 within the normal range were associated with an increased risk of developing CKD, a rapid decline in GFR, and an increased risk of complications (11). In euthyroid patients with diabetic nephropathy, elevated TSH and FT4 were associated with decreased eGFR in diabetic patients (12). A larger cohort with longer follow-up is needed to further verify the relationship between thyroid hormones and IgAN.

Similar to the relationship between hypothyroidism and CKD, lower FT4 may contribute to the progression of renal function in IgA nephropathy through the following aspects (1). Hemodynamic changes: Lower FT4 cause decreased cardiac contractility, decreased circulating blood volume, and renal hypoperfusion (13). Besides, lower FT4 reduce the synthesis of vascular endothelial relaxation factors, leading to arterial stiffness and increased systemic vascular resistance (14). In addition, the sensitivity to β-adrenoceptor is decreased in patients with lower FT4, and the expression and release of renin are decreased, resulting in the decreased activity of the renin-angiotensin-aldosterone system (RAAS) (2, 15). Glomerular changes: Lower FT4 lead to adaptive preglomerular vasoconstriction caused by filtrate overload due to inadequate reabsorption of sodium and water in the proximal tubule. This reduces renal blood flow, leading to prerenal renal injury (16). On the other hand, lower FT4 cause changes in glomerular structure, such as glomerular basement membrane thickening or mesangial matrix widening (17), which further reduces renal blood flow (3). Tubular changes: Lower FT4 regulate renal water and electrolyte metabolism mainly by affecting urine concentration and dilution and renal tubular reabsorption (18, 19). The above mechanisms synergistically lead to the progression of renal function. In addition, Benedetti et al.’s preclinical studies have shown that alterations in thyroid hormones are critical in podocyte pathology associated with diabetic nephropathy (20, 21). They identified thyroid hormone receptor α1 (TRα1) as a key regulator of DN pathogenesis. Although TRa1 has not been studied in IgA nephropathy, it could be an interesting mechanism to clarify in the future.

This study showed that serum creatinine is another independent risk factor for renal composite outcomes in patients with IgAN. The consistency between serum creatinine and renal function is close to poor prognosis of patients.

Renal biopsy is the gold standard for the diagnosis of IgAN, and it can also predict the prognosis of IgAN relatively. As one of the pathological indicators of IgAN, the predictive effect of T on renal prognosis has been widely validated by in previous studies (22). In the univariate Cox regression analysis of this study, T-score was also associated with the prognosis of IgAN. And in the multivariate Cox regression analysis with FT4 as a continuous variable, T2 was still an independent factor for poor prognosis of IgAN. Therefore, we combined FT4 and T-score in order to get more accurate predictive efficacy. The results showed that the combination of FT4 and T-score was the best than these two alone. As mentioned in previous studies, T-score is an independent risk factor for poor prognosis of IgAN, and this study further found that FT4 combined with kidney biopsy can greatly improve the predictive value of patients’ prognosis, which is important for more accurate and comprehensive clinical judgment of the prognosis of IgAN patients.

However, this study still has some limitations. This study is a single-center retrospective study, so the study may suffer from selection bias and a center-specific effect. Further prospective, multi-center studies with a larger sample size and longer follow-up, are needed for validation.

## Conclusion

FT4 was a protective factor for renal progression of IgAN patients. In addition, FT4 combined with tubular atrophy/interstitial fibrosis had a high predictive value for poor prognosis of IgAN.

## Data availability statement

The original contributions presented in the study are included in the article/**Supplementary material**; further inquiries can be directed to the corresponding author/s.

## Ethics statement

The studies involving humans were approved by the Ethics Committee of the Third Affiliated Hospital of Soochow University. The studies were conducted in accordance with the local legislation and institutional requirements. Written informed consent for participation was not required from the participants or the participants’ legal guardians/next of kin in accordance with the national legislation and institutional requirements.

## Author contributions

BY: Data curation, Investigation, Methodology, Software, Writing – original draft. WZ: Data curation, Investigation, Methodology, Software, Writing – original draft. LC: Data curation, Investigation, Writing – original draft. LT: Data curation, Investigation, Writing – original draft. YN: Data curation, Investigation, Writing – original draft. MY: Conceptualization, Funding acquisition, Resources, Supervision, Writing – review & editing. YY: Conceptualization, Funding acquisition, Supervision, Writing – review & editing.
