# Supplementary material for: The predictive value of free thyroxine combined with tubular atrophy/interstitial fibrosis for poor prognosis in patients with IgA nephropathy
Source: Front Endocrinol (Lausanne). 2024 May 14;15:1372824. doi: 10.3389/fendo.2024.1372824 (PMC11130362; doi:10.3389/fendo.2024.1372824)

**Supplementary Table 1** The CKD-EPI equation.

| **Gender** | **Creatinine (mg/dL)** | **Formula** |
| --- | --- | --- |
| female | ≤0.7 | 144×(Scr/0.7)^-0.329^× (0.993) ^age^ |
| female | >0.7 | 144×(Scr/0.7)^-1.209^× (0.993) ^age^ |
| male | ≤0.9 | 141×(Scr/0.9)^-0.411^× (0.993) ^age^ |
| male | >0.9 | 141×(Scr/0.9)^-1.209^× (0.993) ^age^ |

**Supplementary Table 2** Univariate Cox analysis of renal composite outcomes in IgAN patients without thyroid diseases and thyroid hormone replacement therapy.

| **Variable** | **Univariate Analysis** | |
| --- | --- | --- |
|  | **HR (95% CI)** | **P-Value** |
| Gender, female | 1.03 (0.41-2.63) | 0.947 |
| Age, y | 1.01 (0.98-1.05) | 0.488 |
| Hypertension | 2.28 (0.91-5.68) | 0.078 |
| Anemia | 11.40 (4.51-28.83) | <0.001 |
| Hypoalbuminemia | 4.61 (1.74-12.24) | 0.002 |
| Hyperuricemia | 3.36 (1.21-9.36) | 0.020 |
| Scr, μmol/L |  |  |
| 24h urinary protein, g/L | 1.18 (1.05-1.32) | 0.004 |
| T1^a^ | 4.99 (1.34-18.63) | 0.017 |
| T2^a^ | 41.05 (12.04-139.95) | <0.001 |
| C1^b^ | 1.99 (0.71-5.59) | 0.194 |
| C2^b^ | 9.95 (2.70-36.62) | 0.001 |
| TSH, uIU/ml | 0.99 (0.68-1.46) | 0.975 |
| FT3, pmol/L | 0.18 (0.08-0.45) | <0.001 |
| FT4, pmol/L | 0.69 (0.58-0.83) | <0.001 |
| FT4 (≥15.18 pmol/L) | 0.09 (0.03-0.29) | <0.001 |
| ACEI/ARB | 0.37 (0.14-0.98) | 0.037 |
| Prednisone or other immunosuppressive agents | 1.52 (0.58-4.02) | 0.398 |

Scr, serum creatinine; T, interstitial fibrosis/tubular atrophy; C, crescents formation; TSH, thyroid stimulating hormone; FT3, free triiodothyronine; FT4, free thyroxine; ACEI/ARB, angiotension converting enzyme inhibitors/angiotonin receptor blocker; ^a^ T0 was used as the reference; ^b^ C0 was used as the reference.

**Supplementary Table 3** Multivariate Cox analysis of renal composite outcomes in IgAN patients without thyroid diseases and thyroid hormone replacement therapy (FT4 was a continuous variable).

| **Variable** | **Multivariate Analysis** | |
| --- | --- | --- |
|  | **HR (95%CI)** | **P-Value** |
| Anemia | 2.52 (0.49-13.07) | 0.270 |
| Hypoalbuminemia | 0.67 (0.06-7.25) | 0.744 |
| Hyperuricemia | 5.94 (0.88-40.35) | 0.068 |
| Scr, μmol/L | 1.02 (1.01-1.04) | 0.001 |
| 24h urinary protein, g/L | 0.84 (0.65-1.10) | 0.203 |
| ACEI/ARB | 0.45 (0.13-1.58) | 0.212 |
| T1^a^ | 2.38 (0.50-11.45) | 0.279 |
| T2^a^ | 15.44 (3.14-75.80) | 0.001 |
| C1^b^ | 1.23 (0.30-4.96) | 0.773 |
| C2^b^ | 14.76 (1.16- 187.66) | 0.038 |
| FT3, pmol/L | 0.81 (0.19-3.52) | 0.780 |
| FT4, pmol/L | 0.63 (0.46-0.85) | 0.003 |

Scr, serum creatinine; T, interstitial fibrosis/tubular atrophy; C, crescents formation; TSH, thyroid stimulating hormone; FT3, free triiodothyronine; FT4, free thyroxine; ACEI/ARB, angiotension converting enzyme inhibitors/angiotonin receptor blocker; ^a^ T0 was used as the reference; ^b^ C0 was used as the reference.

**Supplementary Table 4** Multivariate Cox analysis of renal composite outcomes in IgAN patients without thyroid diseases and thyroid hormone replacement therapy (FT4 was a categorical variable).

| **Variable** | **Multivariate Analysis** | |
| --- | --- | --- |
|  | **HR (95%CI)** | **P-Value** |
| Anemia | 1.05 (0.22-5.10) | 0.950 |
| Hypoalbuminemia | 1.76 (0.19-16.63) | 0.623 |
| Hyperuricemia | 9.86 (1.33- 73.12) | 0.025 |
| Scr,μmol/L | 1.02 (1.01-1.04) | <0.001 |
| 24h urinary protein, g/L | 0.76 (0.53-1.07) | 0.117 |
| ACEI/ARB | 0.68 (0.21-2.16) | 0.512 |
| T1^a^ | 3.82 (0.77-19.06) | 0.102 |
| T2^a^ | 14.53 (2.90-72.87) | 0.001 |
| C1^b^ | 2.05 (0.49-8.55) | 0.325 |
| C2^b^ | 54.84 (2.26-1331.58) | 0.014 |
| FT3, pmol/L | 0.49 (0.11-2.24) | 0.360 |
| FT4 (≥15.18 pmol/L) | 0.03 (0.01-0.24) | 0.001 |

Scr, serum creatinine; T, interstitial fibrosis/tubular atrophy; C, crescents formation; TSH, thyroid stimulating hormone; FT3, free triiodothyronine; FT4, free thyroxine; ACEI/ARB, angiotension converting enzyme inhibitors/angiotonin receptor blocker; ^a^ T0 was used as the reference; ^b^ C0 was used as the reference.

**Supplementary Table 5** ROC curve analysis of the probability in predicting renal composite outcomes in IgAN patients without thyroid diseases and thyroid hormone replacement therapy.

| **Variable** | **AUC** | **P-value** | **Sensitivity (%)** | **Specificity (%)** |
| --- | --- | --- | --- | --- |
| FT4 | 0.773 | <0.001 | 84.21 | 68.21 |
| T-score | 0.814 | <0.001 | 78.95 | 75.72 |
| T-score+FT4 | 0.876 | <0.001 | 78.95 | 86.71 |

**Supplementary Figure 1** ROC curves of the probability of FT4 and T-score in predicting renal composite outcomes in IgAN patients without thyroid diseases and thyroid hormone replacement therapy.


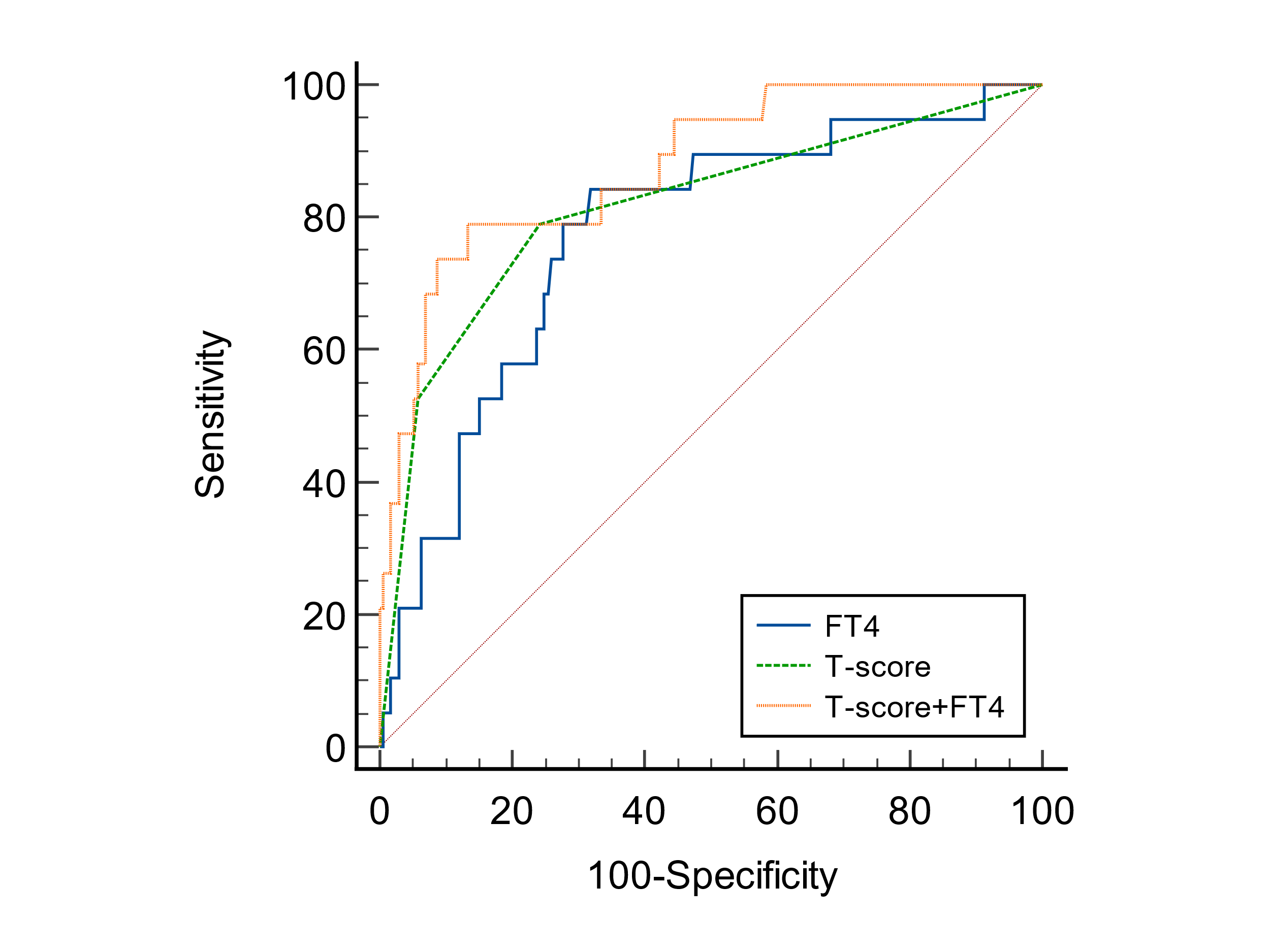

Supplement: Supplementary file 1 [file DataSheet_1.docx]
